# Combination of Fe(OH)_3_ modified diatomaceous earth and qPCR for the enrichment and detection of African swine fever virus in water

**DOI:** 10.3389/fvets.2022.1045190

**Published:** 2022-12-23

**Authors:** Hao Wu, Zihan Tian, Lun Yao, Ahmed H. Ghonaim, Xiaoyu Chen, Shengnan Ruan, Huimin Li, Wentao Li, Qigai He

**Affiliations:** ^1^State Key Laboratory of Agricultural Microbiology, College of Veterinary Medicine, Huazhong Agricultural University, Wuhan, China; ^2^The Cooperative Innovation Center for Sustainable Pig Production, Huazhong Agricultural University, Wuhan, China; ^3^Desert Research Center, Cairo, Egypt

**Keywords:** modified diatomaceous earth, Fe(OH)_3_ colloid, virus enrichment, African swine fever virus, waterborne viruses

## Abstract

Water is one of the primary vectors for African swine fever virus (ASFV) transmission among swine herds. However, the low concentrations of ASFV in water represent a challenge for the detection of the virus by conventional PCR methods, and enrichment of the virus would increase the test sensitivity. In this study, aiming to enrich ASFV in water quickly and efficiently, a rapid and efficient water-borne virus enrichment system (MDEF, modified diatomaceous earth by ferric hydroxide colloid) was used to enrich ASFV in water. After enrichment by MDEF, conventional real-time PCR (qPCR) was used for ASFV detection. ASFV were inactivated and diluted in 10 L of water, of which 4 mL were collected after 60 min treatment using the MDEF system. Two thousand five hundred times reduction of the sample volume was achieved after enrichment. A high adsorption rate of about 99.99 (±0.01)% and a high recovery rate of 64.01 (±10.20)% to 179.65 (±25.53)% was achieved by using 1g modified diatomaceous earth for 10 L ASFV contaminated water. The limit of qPCR detection of ASFV decreased to 1 × 10^−1.11^ GU ml^−1^ (genomic units per milliliter) from 1 × 10^2.71^ GU ml^−1^ after concentrating the spiked water from 10 L to 4 ml. Preliminary application of MDEF allowed successful detection of African swine fever virus (ASFV), porcine circovirus type 2 (PCV2), and pseudorabies virus (PRV) in sewage. Thus, the combination of modified diatomaceous earth and real-time PCR is a promising strategy for the detection of viruses in water.

## 1. Introduction

Pathogens in drinking water cause significant hazards to both human and animal health. The causative agents of waterborne disease fall into three major categories, namely, bacteria, viruses, and parasites (1). In 1993, Charles N. Haas estimated that human have a 5% lifetime risk of death from exposure to waterborne viruses (2) and these risks have not changed significantly over time (3). Most pathogens spread through media contaminated by infected animals' body fluids, exhaled aerosols, and fecal or urinary excretions. Many viruses are detected in water, including environmental waters, bath water, river, and seawater (4). The use of sedimentation, filtration, and other sanitization methods have decreased the risk of infection by pathogens in human drinking water (5). However, relatively little research has focused on the risk assessment of pathogens present in water used for animal production. Most livestock farms use untreated or inadequately treated river- or groundwater, posing a high risk of diseases to livestock and threatening food safety. P.F.M. Teunis reported that traditional water treatment methods, such as long-term storage, flocculation/precipitation, filtration, and ozone disinfection, cannot fully disinfect water, and the low concentration of pathogens in post-treatment samples frequently result in zero counts during measurement (6).

Most current assessment procedures for water quality and disease risk focus of the water's bacterial CFU index. However, there is no association between bacterial indicators and the type and number of waterborne viruses, and consequently, waterborne viruses are often ignored (7). Hence, efficient and cost-effective enrichment methods are urgently needed for the detection of waterborne viruses. Such procedures would allow the assessment of the biosafety risk of water.

Pork is a leading source of high-quality protein in many people's diets and, thus, its supply and safety have significant implications for human health. The emergence of several swine viral diseases can potentially cause pork supply shortages and international trade restrictions. In particular, an acute and highly contagious viral disease (mortality rate exceeding 90%), African swine fever (ASF), is currently causing severe economic losses to the swine industry. It is especially serious since ASF was reported to spread in China in 2018, which has half of the world's swine population. ASF has been listed as one of the notable diseases by the World Organization for Animal Health (WOAH) because of its significant economic, trade, and food-security implications (8).

ASF, belonging to the genus *Asfivirus* of the family *Asfarviridae* is a large, enveloped, double-stranded DNA virus. It can be transmitted through different routes, such as direct or indirect contact with infected pigs and their secretions, excretions, blood, tissues, pork, and pork products, as well as being transported in contaminated water, vehicles, feed, personnel, and other approaches (9). Strong biosecurity measurements have been applied on swine farms in ASF-affected areas to prevent the spread of the disease. Even though personnel, vehicles, and goods can be managed, it is difficult to avoid the spread of ASFV to a pig farm if flooding with ASFV-contaminated water occurs. Within a farm, ASFV is spread primarily through virus-infected saliva or feces. Sewage from washing pens, water trough residues (10), and other manufacturing activity could easily lead to ASFV pollution in affected pig farms. ASFV can infect pigs at a dose as low as 1 TCID_50_; therefore, pigs can be easily infected by ASFV-contaminated water (11). As the detection of ASFV at low concentrations is challenging, an extra enrichment step is required to increase the template concentration before virus detection. Pei and colleagues reported that the number of pathogens in river water, well water, and other water sources are extremely low and often more than 10 L of water is required for enrichment for pathogen detection (12).

Viruses and other bio-colloids have a pH-dependent surface charge in polar media such as water. This electrostatic charge determines the mobility of the soft particle in an electric field, governing its colloidal behavior, which in turn plays a key role in viral adsorption processes. The isoelectric points (IEPs) of viruses range from 1.9 to 8.4, with most in the region of 3.5 to 7.0 (13). Viruses can be adsorbed on a solid matrix by electrostatic attraction or hydrophobic interaction at a defined pH value. Because of this electrochemical property, charged filter material can be used for adsorption of viruses in water. The adsorbed virus can then be eluted from the membrane for detection. Two types of filters are used to concentrate viruses, namely, electro-positively charged filters to concentrate viruses at around pH 7.0 (14–16) and electronegatively charged filters to concentrate viruses at lower pH (17). The adsorption efficiency can be further enhanced by modifying the surface charge of the filter with divalent and trivalent cations such as aluminum (Al^3+^), magnesium (Mg^2+^), ferric iron (Fe^3+^), and other ions (18). A combination of charged membrane filters and microfluidic filtration techniques have also been used to process large volumes of water. These methods are particularly useful when processing large sample volumes and can be used on a scale of liters. However, the miniaturization of filtration techniques into microfluidic devices may result in clogging, limiting their applications to clinical samples (1). Since water in natural environments, such as rivers and wells, is usually weakly alkaline (pH > 7), and viruses carry a negative charge on their surfaces (IEP < pH), positively charged filter media are extremely efficient for capturing viruses (15, 16, 19, 20). Seeley and Primrose coated microporous filters with aluminum hydroxide. The filters tended to be clogged, reducing their filtration of water, and thus reduced their application efficacy. Michen et al. reported that modified diatomaceous earth allowed better water flow due to its larger pore size and the fact that viruses may be retained by adsorption mechanisms resulting from intermolecular and surface forces (21). Emerging water treatment technologies using ferrous and zero-valent iron have shown the potential of reducing viral contamination using both inactivation and adsorption. Iron electrocoagulation was investigated for virus mitigation in drinking water using laboratory experiments (22).

Methods such as ultracentrifugation, immuno-filtration (23), immunomagnetic separation (24), precipitation, and organic flocculation (25) have also been used for virus enrichment. According to the recommendations by the manual of Diagnostic Tests and Vaccines for Terrestrial Animals (World Organization for Animal Health, WOAH), real-time PCR is widely used for ASFV detection. A promising method for detecting ASFV in farms could be the combination of an enrichment system and real-time PCR.

## 2. Materials and methods

### 2.1. Preparation of MDEF filter and EGM filter

**Figure 1:**
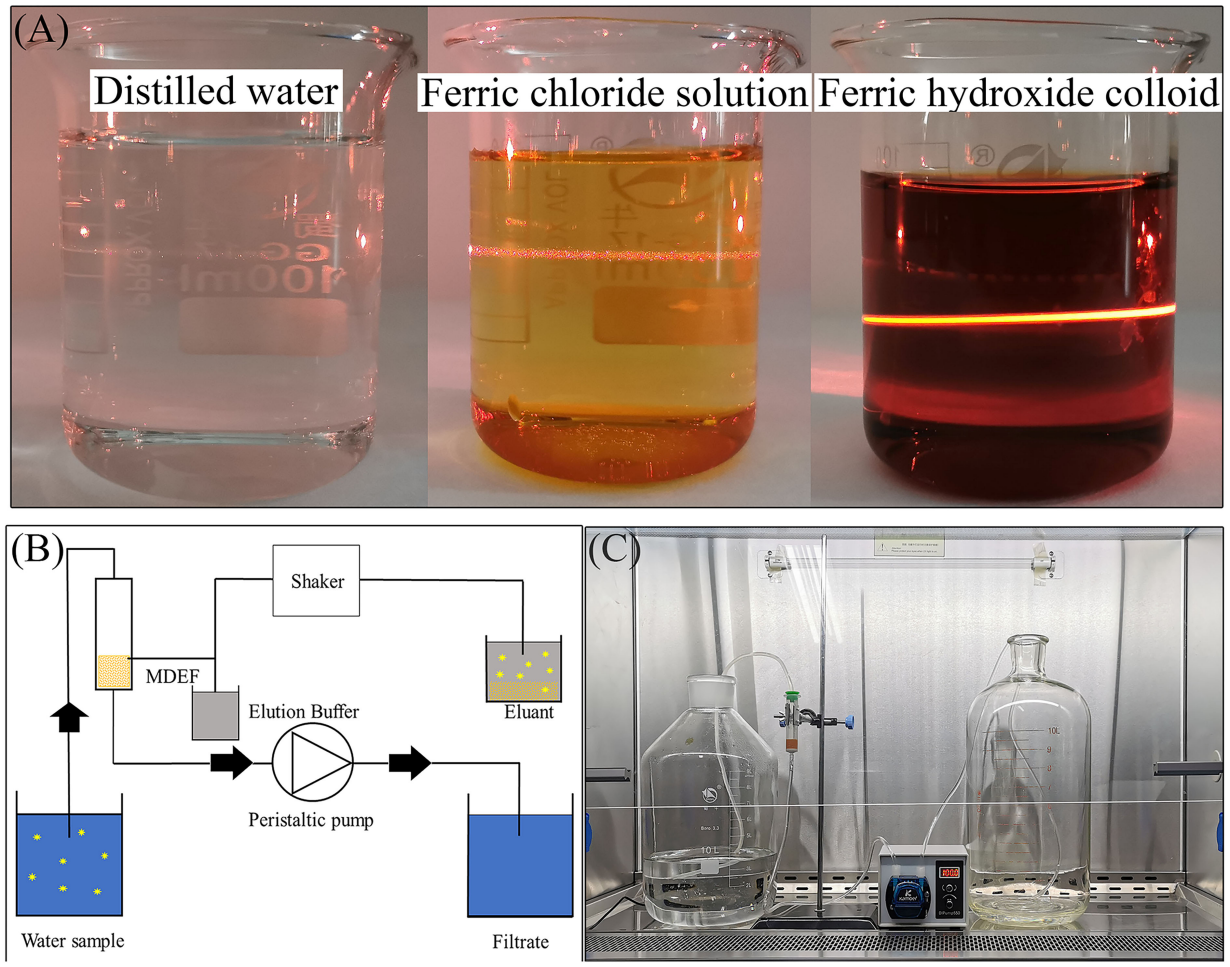
A laser pen was used to irradiate three types of liquid from the side of the beaker, with the appearance of an obvious optical path in the Fe(OH)_3_ colloid which was not apparent in neither the ferric chloride solution nor the distilled water **(A)**, schematic diagram showing the MDEF enrichment and elution procedures **(B)**, and the actual MDEF system **(C)**.

The MDEF system's filter material was diatomaceous earth (Qingdao Ocean Chemical Co., Ltd., Qingdao, China) with Fe(OH)_3_ colloids attached to the surface (Supplementary Figure S1). A saturated solution of FeCl_3_ was prepared by dissolving 1.6 g of ferric chloride hexahydrate (Sinopharm Chemical Reagent Company Limited, Shanghai, China) in 1 ml of distilled water at room temperature (25°C). Next, 0.5 ml of saturated FeCl_3_ solution was added dropwise to 100 ml boiling distilled water. The heating equipment was turned off when the solution turned a burgundy color. After standing for 1 h, the Tyndall effect was applied to assess the development of Fe(OH)_3_ colloids (there is a distinct light channel when the colloid is illuminated by a laser pointer). The pellets should not be visible in this solution (Figure 1A). One hundred grams of diatomaceous earth with a size range of 0.12–0.16 mm were then mixed with 100 ml of the Fe(OH)_3_ colloids, and the mixture was dried at 50°C for more than 24 h. One gram of the dried modified diatomaceous earth was then applied to a polypropylene column (JinYang Filter Equipment, Hebei, China) with an inside diameter of 1.5 cm and a height of 7.5 cm pre-packed with a filter pad (JinYang Filter Equipment, Hebei, China).

The polypropylene filter cartridges and Al(OH)_3_ precipitates were prepared for filter cartridge systems with electropositive granule media (EGM) as previously described (26). First, 1.26 g AlCl_3_ and 8.55 ml of 2 mol L^−1^ Na_2_CO_3_ were used to create an Al(OH)_3_ precipitate. This was mixed well with 80 g silica gel (Marine Chemical Co., Qingdao, China) and dried at 50°C for over 24 h, resulting in the EGM. Lastly, 1 g of the EGM was gently added to a polypropylene filter cartridge containing sterile water (26, 27).

### 2.2. Description of the MDEF system

The MDEF system comprised two water containers, two PVC (polyvinylchloride) pipes with inner diameters of 4.8 mm, an MDEF filter, and one peristaltic pump (Figures 1B, C). Water samples flowed into the collection barrel after passing through the filter column and peristaltic pump (maximum pumping speed of 250 ml min^−1^). After filtration, the ASFV on the MDEF were eluted using elution buffer. Three types of elution buffer (the details are listed in Table 1), including 10× nutrient broth medium (10×NB), 1M NaCl, and 1.5% beef extract with 0.05 M glycine (1.5% GBE), were tested to compare their efficiency for virus elution. Four milliliters of elution buffer were added to the filtration column with the virus for MDEF suspension in the added buffer. The suspension was transferred to 10 ml Eppendorf (EP) tubes, placed on a horizontal shaker, and shaken for 1 h to ensure that the MDEF could release the ASFV into the elution buffer. After 1 h of shaking, the suspension was allowed to precipitate, and 1 ml of the supernatant was used for qPCR analysis.

**Table 1:** Recovery efficiency of three elution buffers at different pH values.

**Elution buffer**	**Ingredients (m/v)**	**ASFV in spiked water (log GU/μL)**	**% Recovered** ±**SD**			
			**pH 3.0**	**pH 5.0**	**pH 7.0**	**pH 9.5**
1M NaCl	1 mol/L NaCl	5.97	0.18 ± 0.00	0.18 ± 0.00	0.18 ± 0.00	0.18 ± 0.00
1.5% GBE	1.5% beef extract with 0.05m glycine		0.18 ± 0.00	1.42 ± 0.97	2.40 ± 3.14	3.93 ± 5.30
10×NB	10% Peptone 3% Beef Extract and 5% NaCl		2.31 ± 1.61	40.01 ± 2.45	71.64 ± 5.23	82.76 ± 3.55

### 2.3. Preparation of spiked water sample

ASFV was inactivated at 60°C for 60 min in a Class II biosafety cabinet in an ABSL-3 laboratory (8). Inactivation was confirmed by inoculation into porcine alveolar macrophages (PAM) cells resulting in no virus growth. Samples were then transferred to a BSL-2 laboratory for follow-up testing. Briefly, inactive anticoagulated blood was subjected to three freeze-thaw cycles and centrifuged at 12,000 rpm to remove cell debris. Varying dilutions of the supernatant were then added to water, resulting in spiked water samples.

### 2.4. Nucleic acid extraction

ASFV DNA was extracted using the TIANamp Genomic DNA Kit (DP304) (TianGen Biotech (Beijing) CO., TD., Beijing, China) according to the manufacturer's instructions. DNA and RNA in clinically samples were extracted simultaneously using the TIANamp Virus DNA/RNA Kit (DP315) (TianGen Biotech (Beijing) CO., TD., Beijing, China). Two hundred microliter samples were used for one extraction. Nucleic acid negative controls were prepared at this stage for each treated and negative control sample by running parallel extractions of nuclease-free water with the kit. The extracted DNA and controls were stored at −20°C until TaqMan^®^ PCR amplification.

### 2.5. TaqMan^®^ PCR amplification

The detection and quantification of 250 bp of the ASFV B646L genes were performed as previously described by King and colleagues (28). This method is recommended by the WOAH. Nuclease-free qPCR Reaction Master Mix (2×) (Takara Bio (China) Co., Ltd.) was prepared in advance. Primers (Sangon Biotech, China) were prepared at a concentration of 10 pmol/μl. Primer F sequence 5′-CTGCTCATGGTATCAATCTTATCGA-3′; Primer R sequence 5′-GATACCACAAGATC(AG)GCCGT-3′. Fluorescent-labeled hydrolysis probe (5′-FAM-CCACGGGAGGAATACCAACCC AGTG-3′-TAMRA, Sangon Biotech, China) was used at a concentration of 10 pmol/μl. The PCR reaction mixture was prepared in sterile 1.5-ml microcentrifuge tubes, as described. The reaction mixture contained: nuclease-free water (5 μl); (2 conc.) 2× PCR reaction master mix (10 μl); primer F (10 pmol, 0.4 μl), primer R (10 pmol, 0.4 μl), fluorescent-labeled probe (10 pmol, 0.4 μl). A further 16.2 μl of PCR reaction mixture was added to each well of an optical reaction plate for the assay and 3.8 μl of the extracted DNA template or blank extraction control was added to each well and covered with a cap. The plate was centrifuged for 1 min in a suitable centrifuge to mix the contents, and PCR amplification was performed on CFX Touch 96-well Real-Time PCR Detection Systems (Bio-Rad, Hercules, CA, USA) with the following parameters: one cycle at 50°C for 2 min; one cycle at 95°C for 10 min; 40 cycles at 95°C for 15 s; 58°C for 1 min (28).

### 2.6. Adsorption experiments

In these experiments, 40 ml of distilled water (*n* = 12, *m* = 3) was spiked with inactivated ASFV to final concentrations of 1 × 10^3.61±0.06^ GU ml^−1^ (genomic units per milliliter), 1 × 10^4.78±0.05^ GU ml^−1^, 1 × 10^6.10±0.07^ GU ml^−1^, and 1 × 10^7.73±0.05^ GU ml^−1^. The spiked water samples were mixed in 50-ml centrifuge tubes with the three types of filter materials [aluminum hydroxide (Al(OH)_3_) colloid modified-diatomite, Fe(OH)_3_ colloid modified-diatomite and unmodified diatomite] and placed on a shaker for 1 h. After shaking for 1 h, the filtered material was allowed to settle to the bottom of the flask for 5 min before 2 ml of the supernatant were transferred to a new centrifuge tube for subsequent experiments. Triplication of 0.2 ml aliquots were removed from the supernatants for detection of the remaining ASFV.

### 2.7. Elution experiment

Previous studies have reported electrostatic interactions between proteins and filter surfaces (29). Three strategies were investigated in this study. The first involved the use of an organic buffer containing a high protein concentration, i.e., 10 times the concentration of the nutrient broth medium (10×NB) for detaching the bound virus on the filter. The second option was the use of chloride ions (1M NaCl solution) to neutralize the charge on the surface of modified diatomite, disrupting the electrostatic attraction between the virus and the filter (18). Beef extract (1.5%) with 0.05 m glycine (1.5% GBE, pH = 9.5) has been frequently used for elution, for instance, for the 1MDS cartridge filters recommended by United States EPA (17). The recovery efficiencies of the three different elutes, i.e., 10×NB buffer, 1M NaCl solution, and 1.5% GBE buffer, were compared. The elution buffers were adjusted to specific pH values (3.0, 5.0, 7.0, or 9.5).

### 2.8. Determination of the detection limit of the MDEF/qPCR combination

The MDEF system enriched the inactivated ASFV in the water. Serial dilutions of ASFV standard plasmid DNA were prepared and used to develop a standard curve for quantification of ASFV by qPCR (Supplementary Figure S2). It was observed that when a low amount virus was added into a large volume of water, the detection limit was lower than the theoretical concentration due to Brownian motion. For example, addition of 1 ml of inactivated ASFV (1 × 10^8.39±0.03^ GU ml^−1^) to 10 L water would result in a detectable ASFV genome of 1 × 10^3.87±0.29^ GU ml^−1^, indicating that if the volume increased by 1 × 10^4.00^ times, the concentration could be reduced by 1 × 10^4.52^ times. Hence, the amount of ASFV genome added into the water was used to calculate the recovery rate instead of the amount detected in the spiked water. In practice, this phenomenon hardly ever occurs in spiked water with high viral concentrations.

After calculating the amount of virus input, different amounts of virus were added to 10 L of water to model the different virus concentrations in spiked water. Six final concentrations in spiked water (1 × 10^−0.33±0.06^ GU ml^−1^, 1 × 10^0.93±0.06^ GU ml^−1^, 1 × 10^2.14±0.01^ GU ml^−1^, 1 × 10^3.24±0.04^ GU ml^−1^, 1 × 10^4.41±0.04^ GU ml^−1^, 1 × 10^5.35±0.07^ GU ml^−1)^ were prepared in 18 barrels. All the spiked water was filtered and eluted. The viral concentration in the eluents was determined, and the limitations of detection (LOD) of the combined MDEF system and qPCR method were calculated.

### 2.9. PEG precipitation

Polyethylene glycol (PEG) is frequently used to enrich viruses. The capacity of PEG-6000 to precipitate viruses was also evaluated in this study. Different concentrations of PEG-6000 were mixed with 10×NB elution buffer to prepare 10 ml mixtures with 10^4.17±0.01^GU of inactivated ASFV. The solutions were placed in 15-ml centrifuge tubes, mixed well, and incubated at 4°C for 12 h. After centrifugation for 1 h, 9.6 ml of the supernatant was removed, and the precipitate was rinsed with the the remaining 0.4 ml of the supernatant and analyzed by qPCR.

### 2.10. Statistical analysis

Each experiment was performed at least three times. The results were statistically analyzed, and the significance of the differences was determined with a one-way analysis of variance (ANOVA) and Tukey's multiple comparison tests. In all cases, a value of *p* < 0.05 was deemed a significant difference. The adsorption rate was determined by dividing the total number of ASFV genomes in the filtered water by that in the spiked water. The recovery rate was calculated by dividing the total number of ASFV genomes in the eluates by that in the spiked water. The quantitative detection of ASFV nucleotide acid in water samples and eluted solutions was done by qPCR (the standard curve for ASFV B646L gene plasmids was shown in Supplementary Figure S2). The following formulas were used to calculate the adsorption and recovery rates:


$$\begin{array}{l} \text{Adsorption rate }{\left(\%\right)}^{1*}=\;\left(1\;-\;\frac{C_{1}}{C_{0}}\right)\;\times \;100\\ \text{Adsorption rate }{\left(\%\right)}^{2*}=\\ \;\left(1\;-\;2^{CT^{*}before\;absorbed-CT\;after\;absorbed}\right)\;\times \;100 \end{array}$$

Where, *C*_1_ represents the concentration of the ASFV genome left in the water after being absorbed, and *C*_0_ represents the concentration of the ASFV genome in water before being absorbed. CT represents the cycle threshold value determined by qPCR. The adsorption rate was calculated using two formulae. Formula 1^*^ (which was used in this study) could be applied regardless of the quantitative method used. Formula 2^*^ (which is more convenient) can be used when with qPCR quantification and its amplification efficiency was 100% (±5%). The calculated adsorption rates did not differ between the two formulae.


$$\begin{array}{l} \text{Recovery rate }\left(\%\right)\;=\;\frac{Q_{1}}{Q_{0}}\;\times \;100 \end{array}$$

Here, *Q*_1_ and *Q*_0_ represent the quantity of ASFV genome measured in the final eluate after concentration and the quantity of ASFV genome seeded into the spiked water samples before concentration, respectively.

## 3. Results

### 3.1. Adsorption of metal hydroxide colloid modified diatomaceous earth

**Figure 2:**
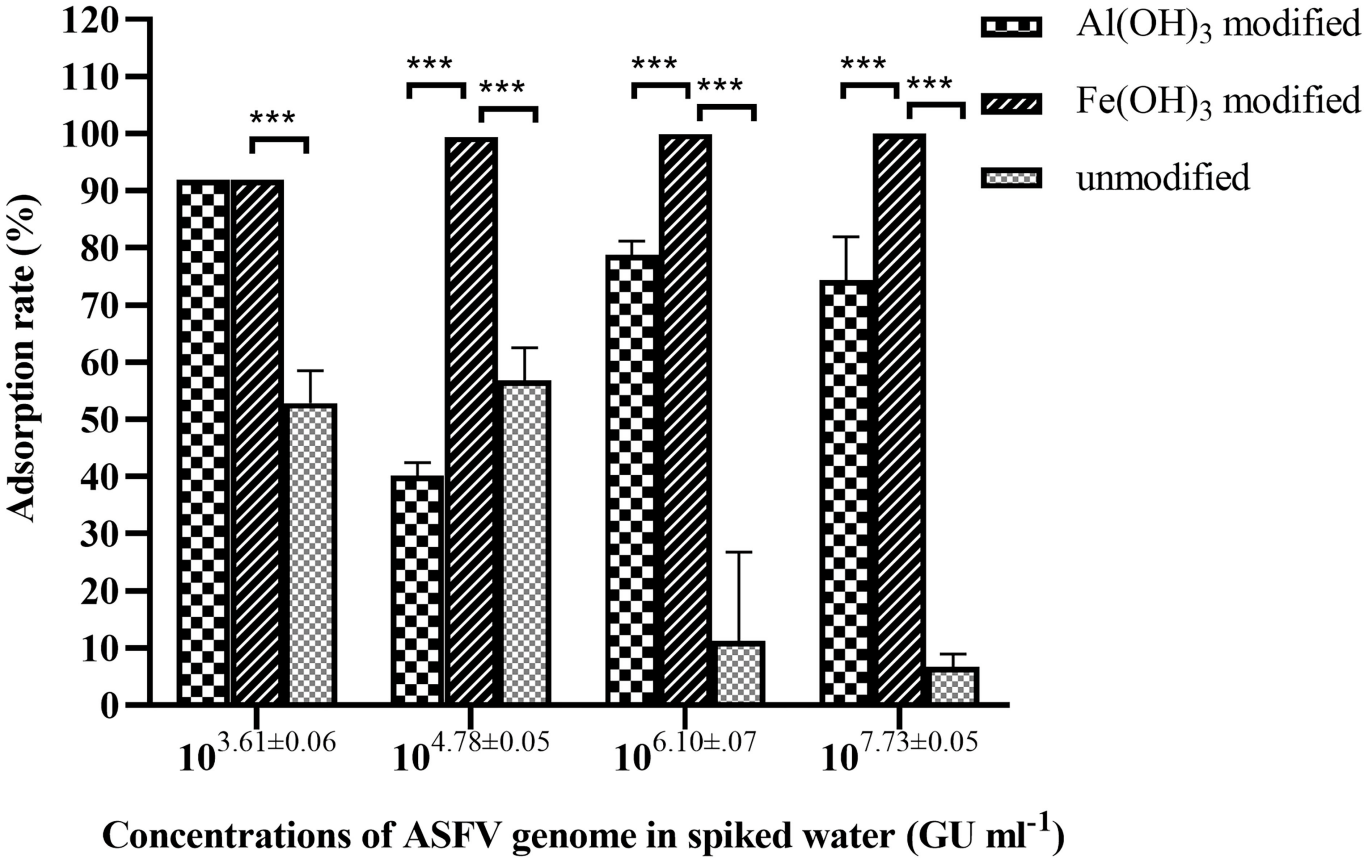
Adsorption of ASFV by Al(OH)_3_-modified, Fe(OH)_3_-modified, and unmodified diatomaceous earth from 40 ml of spiked water containing different concentrations of virus. The adsorption efficiency of the Fe(OH)_3_-modified diatomaceous earth was significantly higher than that of the other two materials. ***Significant difference between groups (*p* < 0.01).

Modification with different salts led to an increase in the zeta potential of the diatomaceous earth (30). The activity of Al(OH)_3_ colloid and Fe(OH)_3_ colloid modified-diatomite were compared with unmodified diatomite to examine their ASFV adsorption capabilities. Each combination was set up with three duplicates to calculate the standard deviation. Figure 2 demonstrates the filter media's adsorption efficiency at various viral concentrations. No genome was detected in the samples with low ASFV concentration (1 × 10^3.61±0.06^ GU ml^−1^) treated by Al(OH)_3_ colloid modified-diatomite and Fe(OH)_3_ colloid modified-diatomite. The CT values of samples that could not be detected (no CT value) were determined as 40 cycles (CT = 40) for calculation. Due to the constraint in the calculation method, the real adsorption rate was higher than the calculated value of 91.93 (±0.003)%.

ASFV nucleic acids were detected at a concentration of 1 × 10^3.28±0.04^ GU ml^−1^, with only a 52.89 (±4.61)% adsorption rate in the unmodified diatomite. The adsorption rates were 6.71 (±1.86) and 11.29 (±12.64)% in the ASFV genome-concentrated spiked water. The Al(OH)_3_ colloid-modified diatomite showed adsorption rates in the range of 91.93 (±0.00) to 40.19 (±1.87)%. The adsorption efficiency of the Fe(OH)_3_ modified diatomite was almost 100%, and no ASFV genome was detected in water after adsorption, even at the highest concentration of ASFV in the spiked water. In general, 1 g of Fe(OH)_3_ colloid-modified diatomite could completely absorb the ASFV in 40 ml water with a concentration <1 × 10^7.73±0.05^ GU ml^−1^. This result is consistent with the findings of Farrah and colleagues (30).

The results indicated that the adsorption efficiency of diatomite modified by Fe(OH)_3_ colloid was much higher than that of the other filter media (*p* < 0.01). Thus, the Fe(OH)_3_ colloid was used as the filter material in the MDEF system.

### 3.2. The recovery efficiency of eluents at different pH conditions

It was found that ASFV absorbed by modified diatomite were effectively eluted using 10×NB (Table 1). The recovery efficiency of the alkaline medium (82.76 ± 3.55% at pH 9.5, 71.64 ± 5.32% at pH 7.0) was much higher than that of the acidic medium (40.01 ± 2.45% at pH 5, 2.31 ± 1.61% at pH 3). However, there was no significant difference in recovery efficiency between pH 7.0 and 9.5. To avoid adjustment of the pH, 10×NB of pH 7.0 was used in subsequent experiments. A non-significant elution of ASFV (0.00 ± 0.00% to 3.93 ± 5.30%) was observed using 1M NaCl and 1.5% GBE as eluents.

### 3.3. Comparison of virus-enrichment methods

**Figure 3:**
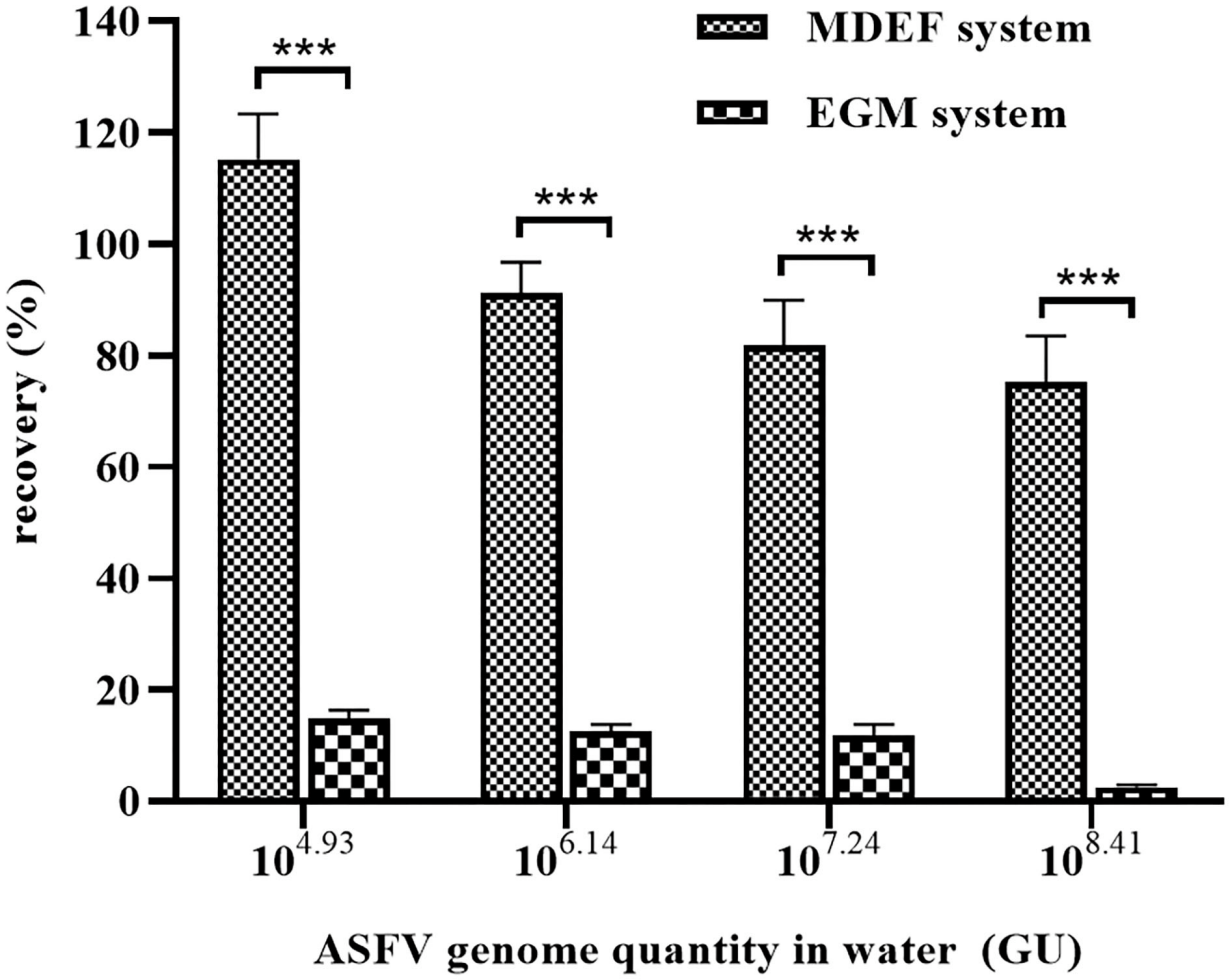
The EGM and MDEF systems were used to recover viruses from spiked water (10 L) containing different ASFV genome concentrations. The recovery rates of the MDEF system were significantly higher than those of the EGM system for all four types of spiked water. ***Significant difference between groups (*p* < 0.01).

Al(OH)_3_ is commonly used for virus enrichment from water (31–33). Recovery of the MDEF was compared to that of the Al(OH)_3_-modified EGM filter cartridge system. The preparation of Al(OH)_3_ colloid-modified diatomite and filtration procedure were based a previously published protocol (26). The recovery of the MDEF system (112.46 ± 16.10%) was significantly higher than that of the EGM (14.71 ± 1.36%) in the recovery of ASFV genomes from 10 L of spiked water (1 × 10^4.93^ GU) with 1 g of filter material. The recoveries of the MDEF and EGM systems declined as the concentration of ASFV genome increased. However, at all concentrations of spiked water, the recovery of the MDEF system was significantly more efficient (*p* < 0.01) than that of the EGM system (Figure 3).

### 3.4. Detection limit of the MDEF/qPCR combination

**Figure 4:**
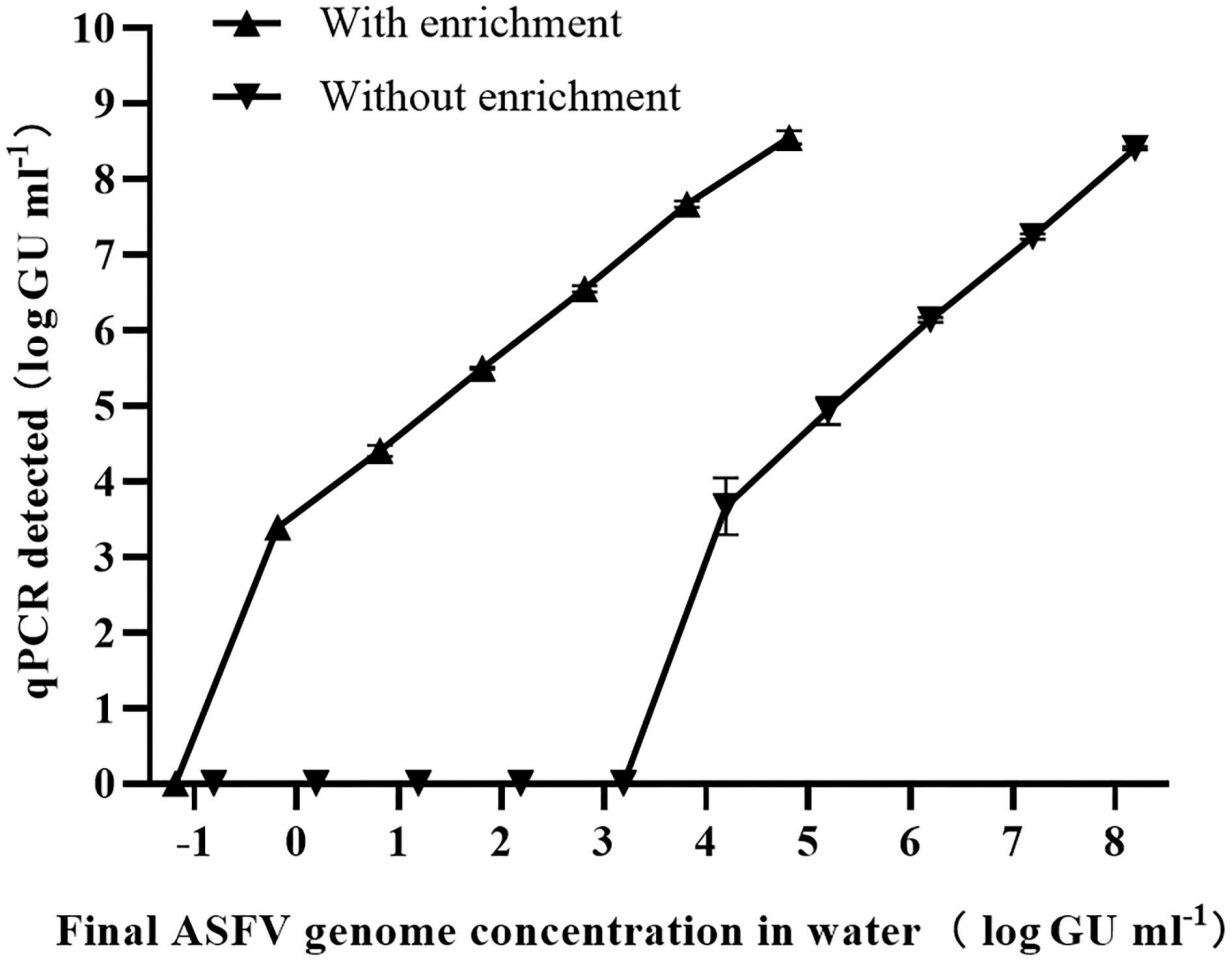
Eighteen barrels of spiked water with six final concentrations of ASFV (10 L per barrel) were enriched using the MDEF system, and the presence of the ASFV genome in both spiked water (without enrichment) and eluates (with enrichment) was measured by qPCR. The LODs were determined for both conditions with the LOD of the 10-L ASFV-contaminated increasing 1 × 10^4.0^ times (from 1 × 10^3.67±0.27^ GU ml^−1^ to 1 × 10^−0.33±0.27^ GU ml^−1^) after enrichment.

The limitations of detection (LODs) of the individual ASFV qPCR and the combined method were used to determine the efficiency of the combined MDEF/qPCR system (Figure 4). The lowest detectable concentrations were 1 × 10^3.67±0.27^ GU ml^−1^(spiked water) and 1 × 10^4.00±0.06^ GU ml^−1^ (eluant). 179.65 (±25.53)% ASFV genome was recovered from the spiked water (1 × 10^−0.33±0.27^ GU ml^−1^) by eluting (1 × 10^4.00±0.06^ GU ml^−1^) after concentrating 2,500 times of the volume of them. These performances demonstrated the efficiency of the system's recovery capacity. The ASFV genome could not be detected in a series of spiked water samples < 10^3.67±0.27^ GU ml^−1^. However, after enrichment, ASFV genome concentrations were detected in eluants as 1 × 10^8.55±0.07^ GU ml^−1^ (64.01±10.20%), 1 × 10^7.67±0.04^ GU ml^−1^ (76.85±6.60%), 1 × 10^6.55±0.04^ GU ml^−1^ (81.84±6.60%), 1 × 10^5.50±0.01^ GU ml^−1^ (91.12±2.31%), 1 × 10^4.41±0.06^ GU ml^−1^ (112.46±16.10%), and 1 × 10^3.40±0.06^ GU ml^−1^ (179.65±25.53%). Their corresponding final concentrations in spiked water were 1 × 10^5.35±0.07^ GU ml^−1^, 1 × 10^4.41±0.04^ GU ml^−1^, 1 × 10^3.24±0.04^ GU ml^−1^, 1 × 10^2.14±0.01^ GU ml^−1^,1 × 10^0.93±0.06^ GU ml^−1^, and 1 × 10^−0.33±0.06^ GU ml^−1^.

These results indicate that the LOD in 10 L of ASFV-contaminated water increased by 1 × 10^4.0^ times (from 1 × 10^3.67±0.27^ GU ml^−1^ to 1 × 10^−0.33±0.27^ GU ml^−1^) using the combined MDEF and qPCR method.

### 3.5. Additional experiments

**Figure 5:**
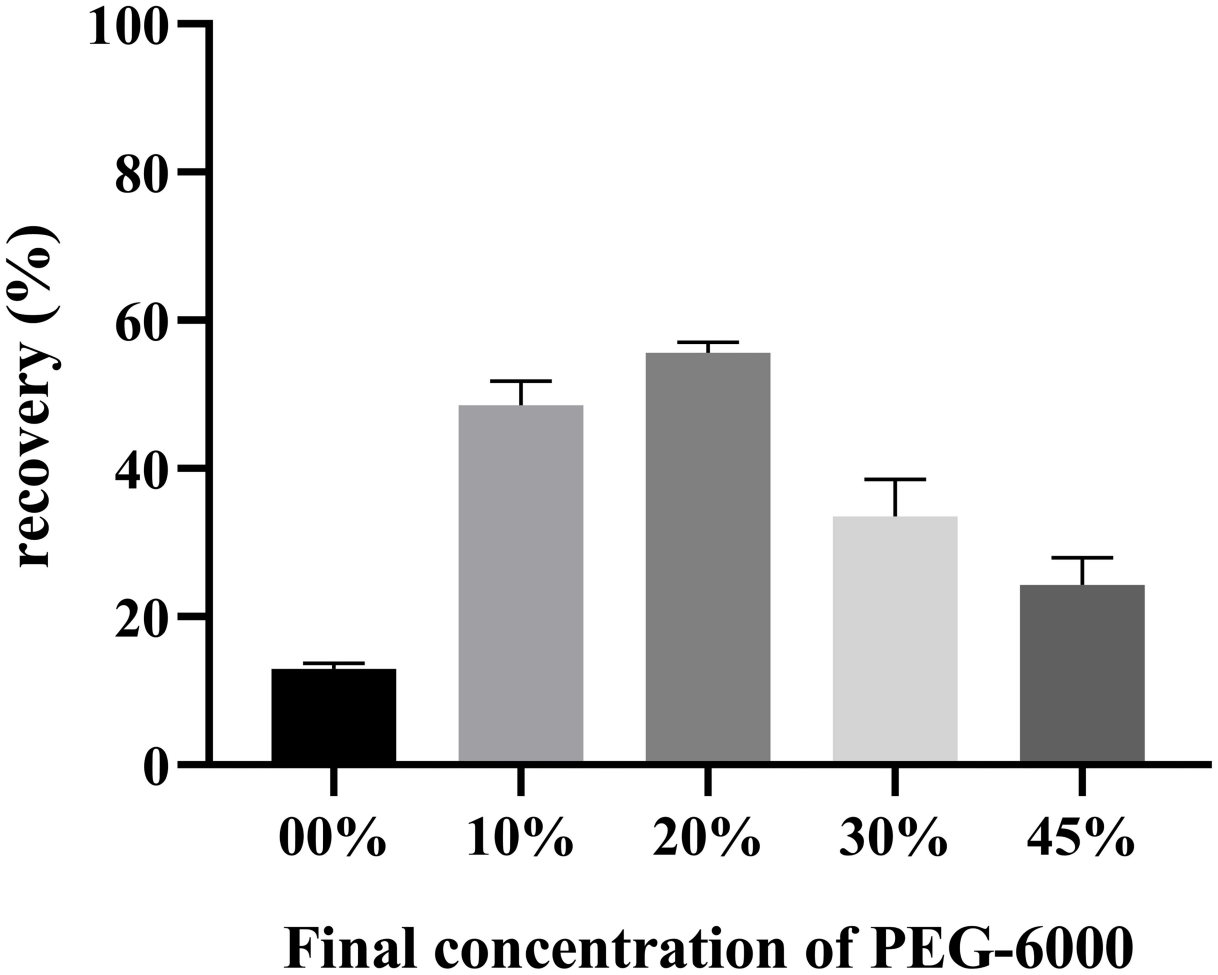
Different final concentrations of PEG-6000 was added to the ASFV-containing eluate for determination viral recovery. After precipitation and centrifugation, the volume of liquid was reduced from 10 ml to 0.4 ml. More than 50.42 (±4.53)% of the virus was recovered using the 20% PEG-6000 solution.

Over 50.42 (±4.53)% of the virus (1 × 10^3.93±3.93^ GU) was recovered using 20% PEG-6000 solution (Figure 5). Although the concentration was 10 times higher than non-PEG-6000 precipitation protocol, this required more than 13 h of treatment and the use of a high-speed centrifuge. Hence, extra treatment was only recommended in well-equipped laboratories.

### 3.6. Natural water experiments

The MDEF system was used to measure a total of 59 samples of natural water and sewage to determine its clinical applications (Table 2). The 59 samples consisted of 10 fecal sewage samples (No. 1–4) from the ASFV animal infection experiments, eight samples from washed pigsties in ASFV-infected farms (No. 5 to 10) where the pigsties had had an ASF outbreak but had since been cleaned and dried and five liters of water were used for sample collection on equipment surfaces through repeated washing of the surfaces, nine samples from unwashed pigsties (No. 11–19) on ASFV-positive farms where the pigsties were undisinfected or disinfected with NaOH and contained lots of sewage, two water samples from a slaughterhouse depilation tank (No. 20 and 21), and 14 samples from well water obtained from five pig farms well water samples from 5 pig farms (No. 31). ASFV was detected in several of these samples. It was notable that some of these samples were diagnosed as ASFV-positive after processing with the MDEF system, whereas they were misdiagnosed as ASFV-negative when only using qPCR for detection.

**Table 2:** The MDEF system was applied to 59 samples of natural water or sewage.

**Sources**	**No**.	**Numbers**	**Volume**	**Virus species**	**CT value**	
					**Without MDEF**	**With MDEF**
Fecal sewage from animal infection assay	1	1	500 ml	ASFV	NT^*1^	36.56
	2	1	200 ml	ASFV	38.62	35.75
	3	1	1 L	ASFV	33.78	30.4
	4	7	500 ml−1 L	-	NT	NT
Water from the clean-washed pigsties	5	1	5 L	ASFV	NT	36.90
	6	1	5 L	ASFV	NT	37.49
	7	1	5 L	ASFV	NT	35.98
	8	1	5 L	ASFV	NT	34.24
	9	1	5 L	ASFV	NT	36.60
	10	3	5 L	–	NT	NT
Water from the unwashed pigsties	11	1	450 ml	ASFV	36.90	36.94
	12	1	400 ml	ASFV	37.22	34.84
	13	1	500 ml	ASFV	39.84	35.45
	14	1	400 ml	ASFV	37.04	36.85
	15	1	1 L	–	NT	32.67
	16	1	450 ml	–	NT	35.38
	17	1	900 ml	ASFV	37.99	31.71
	18	1	900 ml	ASFV	37.06	30.92
	19	1	350 ml	–	NT	NT
Water from slaughterhouse depilation tanks	20	1	5 L	ASFV	NT	35.28
	21	1	600 ml	–	NT	NT
Drinking water from pig farms	22	1	400 ml	PCV2	NT	37.39
Fecal sewage from pig farms	23	1	400 ml	PCV2e^*2^	33.78	29.54
	24	1	400 ml	PRV	NT	38.29
				PCV2d^*3^	30.75	27.78
	25	1	400 ml	–	NT	NT
Sewer ditch from pig farms	26	1	500 ml	PCV2	NT	38.42
	27	1	500 ml	PCV2	NT	35.05
	28	1	500 ml	PCV2	NT	35.51
	29	1	250 ml	PCV2	NT	38.07
	30	2	500 ml	–	NT	NT
Well water from 5 pig farms	31	14	500 ml−5 L	–	NT	NT
Lake water	32	3	10 L	–	NT	NT
Yangtze water	33	3	10 L	–	NT	NT

^*1^ No positive result.

^*2,*3^ Sequencing confirmed that these porcine circoviruses were gene types 2e and 2d.

In addition, the MDEF system was used in the flood-affected pig farms (No. 22–30, Henan province, July 2021), Yezi Lake (No. 32), and Yangtze River (No. 33, Hubei Province, February 2022). Despite the use of small volumes of water, PCV2 and PRV were successfully detected.

## 4. Discussion

Methods for the concentration and enrichment of waterborne viruses have been studied for a while, and many adsorbent materials have been developed. Negatively charged filters require the addition of multivalent salts and acidification of the water sample for efficient virus adsorption, making large-volume sampling difficult; these filters include the Millipore membrane filter (cellulose nitrate) (34) and the Filterite pleated cartridge filter (epoxy-fiberglass) (35). In contrast, positively charged filters require no preconditioning of samples and can concentrate viruses from water over a wider pH range than electronegative filters (18). These materials, however, cannot be widely used in veterinary diagnosis due to the need for expensive equipment, inefficient adsorption rates, and differences in virus species. Metal-based adsorption materials have been extensively investigated, especially positively charged filters (15, 18, 24, 27, 36–39). In this study, Fe(OH)_3_-modified diatomaceous earth was found to possess superior adsorption and recovery efficiency than Al(OH)_3_-modified diatomite in the ASFV enrichment experiments. This result can be attributed to the chemical characteristics of these two metal sorbents. According to Luo M, Al^3+^ hydrolysates differed at different pH levels: [Al(OH)_n_]^(n − 3)−^ (*n* = 6, 7, 8, 9, or 10) at pH < 4; [Al_6_(OH)_15_]^3+^, [Al_7_(OH)_17_]^4+^, [Al_8_(OH)_20_]^4+^ and [Al_13_(OH)_34_]^5+^ at 4 < pH < 6; [Al(OH)_3_] at 6 < pH < 8; [Al(OH)_4_]^−^, [Al_8_(OH)_26_]^2−^ at 8 < pH (40, 41). Different hydrolysates exhibit different electrical properties, and Al(OH)_3_ is not positively charged in natural water as a result of its hydrolysates at 7 < pH. Thus, water samples require adjustment to pH ≤ 6.0 before concentration with an aluminum-based method (31, 33). Previous studies have shown that phosphate removal by aluminum-loaded shirasu-zeolite was 80–40% at pH values from 2 to 11 (42). These studies confirmed that poor adsorption effects of aluminum-based methods in neutral or alkaline media. Similar to Al^3+^ hydrolysates, Fe^3+^ hydrolysates also differed at different pH values: Fe^3+^ at pH < 2; FeOH^2+^, Fe(OH)^+^, Fe_2_${\text{(OH)}}_{2}^{4+}$, Fe_3_${\text{(OH)}}_{4}^{5+}$ and other polymers at 2 < pH < 8.1; Fe(OH)_3_ at 8.1 < pH < 12; and Fe${\text{(OH)}}_{4}^{-}$ at 12 < pH. Thus, ferric-based materials are positively charged in solutions with pH < 8.1 (40).

The findings of this study indicated that the use of ferric-based materials for the adsorption of negatively charged groups in natural water have stronger electrostatic attraction than aluminum-based materials. Our findings are consistent with previous studies reporting that ferric hydroxide outperforms aluminum hydroxide in the removal of negatively charged groups such as arsenate (43, 44).

We found that the recoveries using the MDEF and EGM systems declined as the ASFV genome concentration increased. A previous study by Armanious et al. investigated the mechanism by which viruses bind to adsorbents (29). These authors found that virus-sorbent interactions were governed by long-ranged electrostatic forces together with contributions from the hydrophobic effect, while the shorter-range van der Waals interactions were of secondary importance. The topographic irregularities on both the virus and sorbent surfaces influenced steric effects. In our study, the long-range electrostatic interactions on MDEF gradually decreased as the amount of adsorbed virus increased, leading to reduced virus adsorption. At the same time, the adsorption of more virus to the MDEF surface leads to steric effects, further weakening the interaction between the MDEF and virus. Thus, the MDEF recovery rate gradually decreased as the virus load increased. Increasing the weight of the filter media could be a solution, but it can only be considered when the volume of water exceeds 10 L.

CD2v (encoded by pE402R) and p12 (encoded by ORF 061R) are the primary adhesion proteins present on the ASFV external envelope membrane (45), and their isoelectric points have been predicted to be 6.21 and 7.63, respectively (https://web.expasy.org/compute_pi). Based on these values, the surface of ASFV was predicted to be negatively charged at pH > 6.21. A critical characteristic of the adsorbents is surface charge, which is expressed as the zeta potential of the adsorbent surface (46). Although the electrostatic force constitutes one of the mechanisms involved in metal-based adsorption, the mechanism of MS2 virus removal by iron coagulation involves the adsorption of negatively charged virus particles onto the positively charged iron oxyhydroxide, FeOOH(s), floc particles, similar to the mechanism proposed for virus removal by the precipitation of aluminum hydroxide in the Standard Methods virus concentration procedure (47–49). The results of a study by Sobsey and Jones (50) supported the idea that electrostatic forces were instrumental in virus–filter interactions due to the correlation between zeta potential (i.e., electrokinetic potential) measured for the electronegative and positively charged adsorptive materials, and the retention efficiencies were measured for each filter. These reports explained the mechanism by which the MDEF system was superior to the EGM system in the process of concentrating ASFV in natural water.

The low recovery of 1.5% GBE in this study can be explained by the strong electrostatic force of the iron hydroxide colloid compared to other filter media, such as nitrocellulose membranes, 1MDS Cartridge filters, and Al(OH)_3_ colloids. The elution buffer (10×NB) had a high protein concentration to dislodge the bound virus from the modified diatomite through competitive binding. While previous studies have demonstrated the use of ferric-based materials in adsorption, these were rarely used in enrichment and recovery, probably due to the use of ineffective eluents (51). Here, the 10×NB buffer was shown to elute viruses from the materials more efficiently compared to other eluents.

Electronegative filters require acidification or the addition of polyvalent salts to water samples before use, which makes large-volume sample processing difficult. Thus, positively charged filter media present an alternative to electronegative adsorbents. Although Virosorb 1MDS demonstrated efficient virus adsorption from various water quality types for both small and large volumes, its high cost reduced the affordability of large-scale applications. Thus, the MDEF system is promising as an inexpensive and effective methodology for monitoring the presence of viruses in water. Also, compared with the commonly used ultracentrifugation-based methods, the MDEF system can be used in smaller and less well-equipped laboratories due to its capacity for large-volume processing without the need for ultracentrifugation.

## 5. Conclusion

Although there are a number of viral enrichment methods, many show poor reproducibility and low recovery and are thus limited in their clinical use. Others are limited by complex procedures and high cost. The MDEF system is the first method used to enrich ASFV in water by modify diatomaceous earth with Fe(OH)_3_ colloid, resulting in an efficient and stable enrichment capacity. Viruses were found to be efficiently eluted from the modified diatomaceous earth using a nutritious broth. This system efficiently enriched ASFV in water. It also showed the following advantages for efficient ASFV detection in water: (1) rapid enrichment of ASFV in more than 10 liters of water from various sources; (2) increased viral concentrations at least 1 × 104 times after enrichment; (3) easy operation; (4) portable and outdoor-friendly; (5) low cost and widely use.

## Data availability statement

The original contributions presented in the study are included in the article/Supplementary material, further inquiries can be directed to the corresponding author/s.

## Ethics statement

The animal study was reviewed and approved by Laboratory Animal Ethics Committee of Huazhong Agriculture University (HZAUSW-2022-0017).

## Author contributions

HW and ZT contributed to the conception or design of the work, and the acquisition of data. HW and LY completed the data analysis. XC, AG, HL, SR, and WL drafted the manuscript and revised it for important intellectual content. QH provided the samples and helped to design the experiments. All authors have read and edited the manuscript.

## Supplementary material

Supplementary Figure 1 Scanning electron micrographs of the surface structure of the diatomaceous earth: **(A–C)** modified diatomaceous earth without Fe(OH)_3_, showing a flat clean surface; **(D–F)** modified diatomaceous earth with Fe(OH)_3_, showing coarsening of the diatomaceous earth surface resulting from attachment of the Fe(OH)_3_ colloids.

Supplementary Figure 2 Standard curve for B646L gene plasmids.
