# Supplementary figures and images for: Combination of Fe(OH)3 modified diatomaceous earth and qPCR for the enrichment and detection of African swine fever virus in water
Source: Front Vet Sci. 2022 Dec 23;9:1045190. doi: 10.3389/fvets.2022.1045190 (PMC9822731; doi:10.3389/fvets.2022.1045190)

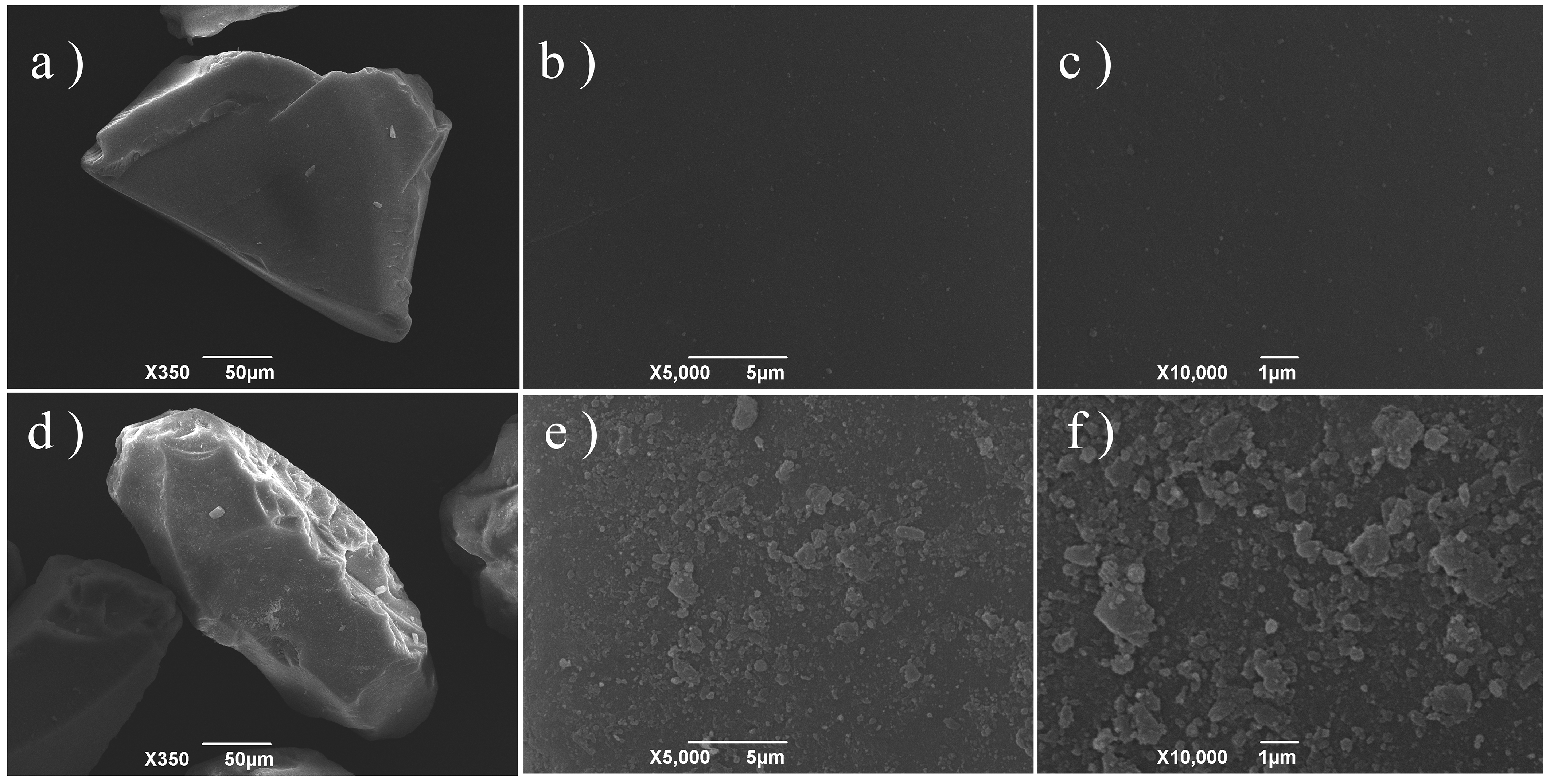

Supplement: Supplementary Figure 1 — Scanning electron micrographs of the surface structure of the diatomaceous earth: (A–C) modified diatomaceous earth without Fe(OH)3, showing a flat clean surface; (D–F) modified diatomaceous earth with Fe(OH)3, showing coarsening of the diatomaceous earth surface resulting from attachment of the Fe(OH)3 colloids. [file Image_1.TIF]

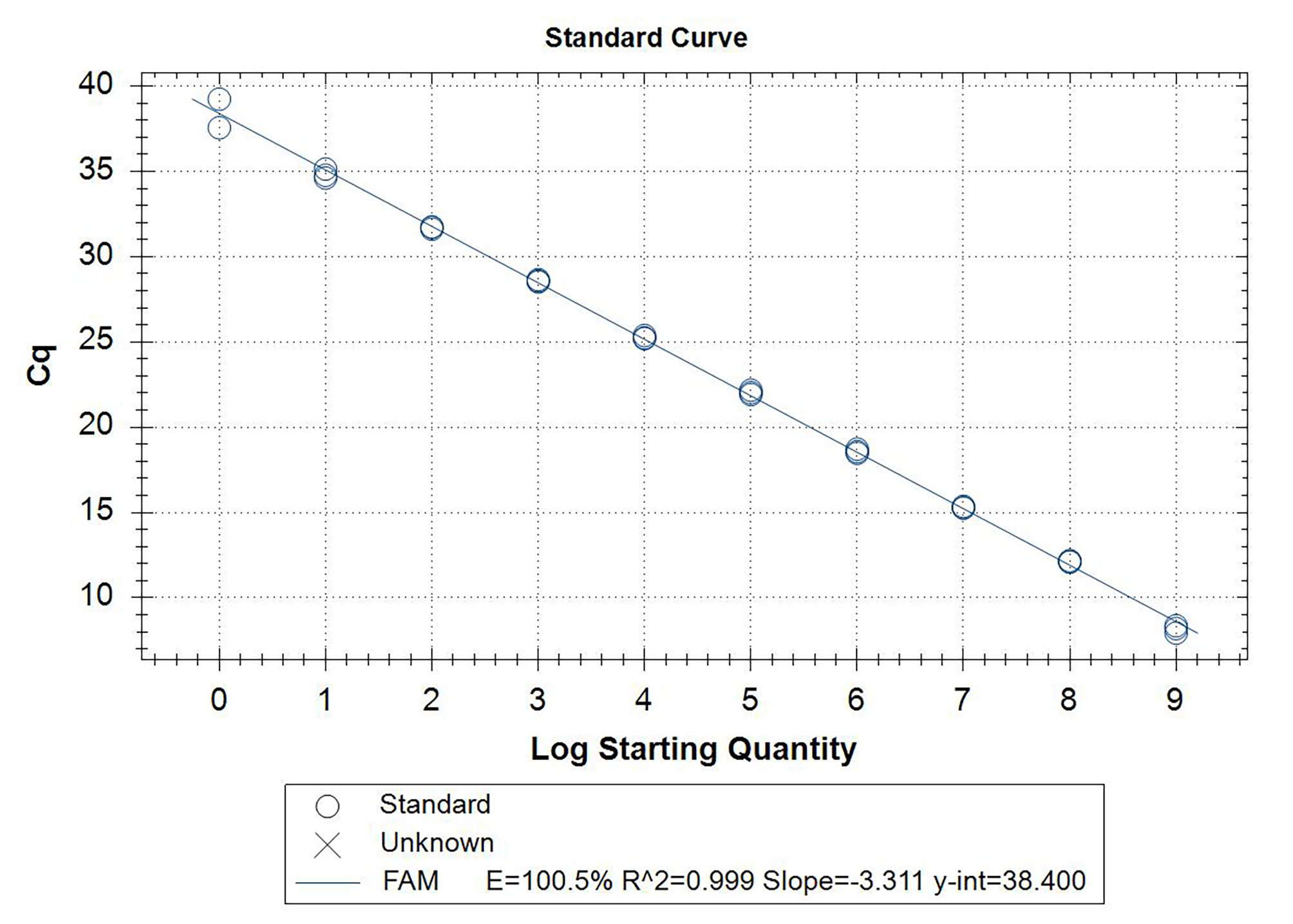

Supplement: Supplementary Figure 2 — Standard curve for B646L gene plasmids. [file Image_2.TIF]
